# Negative association between cognitive functioning and antipsychotic D_2_ receptor occupancy, affinity, and dose after first episode psychosis

**DOI:** 10.1017/S0033291725102900

**Published:** 2026-01-02

**Authors:** Priscilla P. Oomen, Shiral S. Gangadin, Lieuwe de Haan, Franciska de Beer, Oda E. Beune, Doris A.D. Oostendorp, Marieke J.H. Begemann, Nynke Boonstra, Martijn Kikkert, Sanne Koops, Wim Veling, Iris E.C. Sommer

**Affiliations:** 1 Behavioral Science Institute (BSI), Radboud University Nijmegen, Nijmegen, The Netherlands; 2 Center for Clinical Neuroscience and Cognition, University Medical Centre Groningen, University of Groningen, Groningen, The Netherlands; 3 Department of Psychiatry, Amsterdam University Medical Center, University of Amsterdam, Amsterdam, The Netherlands; 4 Department of Research, Arkin Mental Health Care, Amsterdam, The Netherlands; 5 NHL Stenden University of Applied Sciences, Leeuwarden, The Netherlands; 6 KieN VIP Mental Health Care Services, Leeuwarden, The Netherlands; 7 Department of Psychiatry, UMC Utrecht Brain Center, University Medical Center Utrecht, Utrecht, The Netherlands; 8 Department of Psychiatry, University Medical Centre Groningen, University of Groningen, Groningen, The Netherlands

**Keywords:** antipsychotic medication, cognition, dopamine D_2_ affinity, psychosis, schizophrenia

## Abstract

**Background:**

Evidence regarding the effects of antipsychotic medication on cognitive functioning after a first-episode psychosis (FEP) remains inconclusive. This study examined whether dopamine D_2_ receptor occupancy, affinity, and antipsychotic dose are related to cognitive functioning in people in remission from FEP.

**Methods:**

278 remitted FEP participants from the HAMLETT-trial were included. Cognitive functioning was assessed with the Brief Assessment of Cognition in Schizophrenia, 3–6 months after remission. D_2_ receptor occupancy was estimated based on antipsychotic type and dose. Antipsychotics were categorized into partial agonists, or antagonists with high or low D_2_ receptor affinity. Linear regression analyses were performed with inverse probability of treatment weighting to control for differences in characteristics between groups.

**Results:**

D_2_ receptor occupancy was negatively related to global cognition (*β* = −0.18), verbal fluency (*β* = −0.22), and attention and processing speed (*β* = −0.17, all *p* < 0.003). The interaction between daily dose and D_2_ receptor affinity category was significant for global cognition (*p* = 0.0046) and working memory (*p* = 0.0019), but not for verbal fluency after correction for multiple testing (*p* = 0.029). Interactions showed that higher daily dose was related to lower cognitive functioning, with significantly stronger negative effects in high-affinity antagonists compared to other antipsychotics.

**Conclusions:**

The current findings underscore the importance of antipsychotic D_2_ receptor occupancy and affinity for cognitive functioning and suggest better cognitive functioning in users of partial agonists and low D_2_ receptor affinity antipsychotics. This can be important when selecting antipsychotics for individuals with FEP.

## Introduction

People diagnosed with schizophrenia-spectrum disorders (SSDs) are often prescribed antipsychotic medication for at least 1 year to prevent relapse after achieving symptomatic remission. National guidelines are inconclusive as to which antipsychotic drug should be preferred (reviewed in Correll et al., 2022), since differences in efficacy are regarded to be small (Huhn et al., 2019; Schneider-Thoma et al., 2022). Yet, an important reason to prefer one type of medication over another, could be its potential influence on cognitive functioning. Impairments in cognitive functioning are already present very early in the disease and are subject to great variability in those with a first-episode psychosis (FEP) (Catalan et al., 2024; Lee et al., 2024).

Despite extensive research (Baldez et al., 2021; Baldez et al., 2025; McCutcheon, Keefe, McGuire, Marquand et al., 2025; Mishara & Goldberg, 2004), the evidence regarding the effects of antipsychotic medication on cognition remains inconclusive. Large studies performed in FEP (Davidson et al., 2009) and long-term schizophrenia (Keefe et al., 2007) suggest that treatment with antipsychotic medication can have a positive effect on cognitive functioning in SSD (Hou et al., 2020). In these studies, improvements in cognition were shown to be associated with symptom reduction and better general functioning (Helldin, Mohn, Olsson, & Hjärthag, 2020; Johansson, Hjärthag, & Helldin, 2020; Lindgren, Holm, Kieseppä, & Suvisaari, 2020; Santesteban-Echarri et al., 2017). Antipsychotics may therefore indirectly contribute to improve cognitive functioning by reducing psychotic symptoms and increasing general functioning. Achieving symptomatic remission with antipsychotics could also be important for cognitive recovery, as it minimizes the confounding effects of psychotic symptoms (e.g. hallucinations, disorganization). Studying a population of individuals in symptomatic remission therefore allows for a more accurate assessment of cognitive abilities in people with FEP.

On the other hand, antipsychotic maintenance therapy has also been related to negative effects on cognitive functioning, especially at high doses (Allott et al., 2023; Ballesteros et al., 2018; Élie et al., 2010; Husa et al., 2017; Rehse et al., 2016; Woodward, Purdon, Meltzer, & Zald, 2007). Previous studies commonly assessed the effects of antipsychotics on cognition by relating dose equivalents to cognitive functioning (Haddad et al., 2023), by comparing two different drugs (i.e. olanzapine vs. haloperidol) (Désaméricq et al., 2014) or by comparing medication types (i.e. first vs. second generation) (Baldez et al., 2025; Nielsen et al., 2015). Since dopamine receptor binding may play an important role in cognitive dysfunction (Nørbak-Emig et al., 2016; Sala-Bayo et al., 2020), classifying antipsychotic medication based on dopamine D_2_ receptor affinity (i.e. high D_2_ affinity antagonists, low D_2_ affinity antagonists, or partial agonists) may be more useful than classification based on generations of antipsychotics (Zhou, Nutt, & Davies, 2022).

Antipsychotics all share the same mechanism of dopamine D_2_ receptor blockade, yet they vary significantly in their receptor affinity. This dopaminergic binding property has been implicated in the effect of antipsychotics on cognitive functioning (Allott et al., 2023, 2024; Volkow et al., 1998; Yang et al., 2004). Dopamine is an important neurotransmitter in several brain circuits involved in motivation and cognition, such as the reward system, memory, and attention (Goldman-Rakic, 1998). In healthy individuals, administrating antipsychotics results in impaired sustained attention, processing speed, and working memory. This effect was related to the extent of dopamine D_2_ receptor occupancy (Kim et al., 2013; Saeedi, Remington, & Christensen, 2006).

While dopamine D_2_ receptor occupancy, to a certain extent, is effective in treating psychotic symptoms (Uchida et al., 2011), exceeding the optimal dopamine D_2_ receptor occupancy may disproportionally worsen cognitive impairment. Indeed, high dopamine D_2_ receptor occupancy, as measured in positron emission tomography (PET) studies, has been associated with more impaired cognitive functioning in individuals with SSD (Sakurai et al., 2013; Uchida et al., 2009) and subjective worsening of cognitive functioning (De Haan et al., 2000, 2003). Therefore, we may gain a better understanding of the impact of antipsychotics on cognitive functioning by focusing on D2 receptor blockade, in addition to dosage. The D_2_ receptor occupancy of eight antipsychotics can be estimated based on antipsychotic type and dose using meta-analytically derived formulas (Lako et al., 2013). Alternatively, assessing the interaction between antipsychotic doses and categorization of dopamine affinity is possible for all antipsychotics, and may provide comparable insights. These approaches do not require PET scanning and therefore allow evaluation in large samples. Combining both approaches, taking into account occupancy, affinity, and dose of antipsychotic medication, may provide unique and additional insights into the existing literature.

The current study aimed to examine the association between cognitive functioning and dopamine D_2_ receptor occupancy, dopamine D_2_ receptor affinity, and antipsychotic daily dose in a large FEP population in symptomatic remission (i.e. with minimal psychotic symptoms). The current study design avoids two factors that often confound similar studies. First, cognitive functioning is related to (psychotic) symptom severity and general functioning. It is therefore important to study people in symptomatic remission from an FEP, to avoid confounding influences of both active psychotic symptoms and the associated (long-term) treatment thereof. Second, as we analyzed users of different medication types in a non-randomized context, differences in baseline functioning or other characteristics could become confounding factors. To prevent this bias, we used inverse probability of treatment weighting (IPTW) as a statistical solution for patient matching to balance clinical and sociodemographic characteristics between the dopamine D_2_ receptor affinity groups.

## Methods and materials

### Participants

Data were used from the ongoing Handling Antipsychotic Medication: Long-term Evaluation of Targeted Treatment (HAMLETT) study (Begemann et al., 2020). Written informed consent was obtained from all participants and study procedures were performed according to the Declaration of Helsinki (October 2013). Ethical approval was obtained from the research and ethics committee of the University Medical Center Groningen, the Netherlands (EudraCT number: 2017-002406-12). Patients were recruited from 26 outpatient care centers in The Netherlands. Details regarding recruitment and study procedures are described by Begemann et al. (2020). The current study included data from 278 participants aged between 16 and 60 years old who were between 3 and 6 months in symptomatic remission of their first psychotic episode and used antipsychotic medication. Demographic data were collected for all participants with the Comprehensive Assessment of Symptoms and History (Andreasen, 1992) (Table 1). Severity of symptomatology was assessed using the Positive and Negative Syndrome Scale (PANSS; Kay, Fiszbein, & Opler, 1987).

**Table 1.** Demographic and clinical characteristics of the study sample (n = 278)

Characteristics	Value	
Age, median (IQR) in years	25.0	11.0
Sex assigned at birth, male, n/%	195	70.1%
Illness duration, median (IQR) in months	8.0	5.0
Time since remission until cognitive testing, median (IQR) in days	132.0	64.3
Years of education, median (IQR) in years	14.0	1.1
Antipsychotic daily dose (mg olanzapine), median (IQR)	9.5	8.2
Dopamine D_2_ receptor occupancy, median (IQR) in %^a^	65.4	30.9
Type of medication, n/%		
High dopamine D_2_ affinity antagonists,	70	25.2%
*Haloperidol*	27	9.1%
*Flupentixol*	5	1.7%
*Zuclopenthixol*	2	0.7%
*Sulpiride*	5	1.7%
*Amisulpride*	5	1.7%
*Risperidone*	26	8.8%
*Paliperidone*	3	1.0%
*Lurasidone*	2	0.7%
Low dopamine D_2_ affinity antagonists	124	44.6%
*Olanzapine*	112	37.7%
*Clozapine*	4	1.3%
*Quetiapine*	22	7.4%
Partial D_2_ agonists	84	30.2%
*Aripiprazole*	82	27.6%
*Brexpiprazole*	2	0.7%
PANSS, median (IQR)		
*PANSS total*	42.0	14.0
*Positive symptoms*	8.0	3.8
*Negative symptoms*	11.0	6.0
*General symptoms*	22.0	7.0
BACS, mean (SD) Z-score		
*Composite score*	−1.32	1.18
*Verbal memory*	−0.71	1.13
*Working memory*	−0.78	1.13
*Motor speed*	−0.83	1.31
*Verbal fluency*	−1.02	1.10
*Attention and Processing speed*	−1.25	0.90
*Executive function*	−0.16	1.19

Abbreviations: BACS: Brief Assessment of Cognition in Schizophrenia; IQR: Interquartile Range; DDD: Defined Daily Doses; PANSS: Positive and Negative Syndrome Scale; SD: Standard Deviation.

a D_2_ receptor occupancy could not be estimated for n = 16 users of flupentixol, zuclopenthixol, sulpiride, lurasidone, and brexipiprazole.

### Cognitive functioning

Cognitive functioning was assessed using the Dutch version of the Brief Assessment of Cognition in Schizophrenia (BACS; Keefe et al., 2004). The test consists of six subtests that assess different cognitive domains: list learning (verbal memory), digit sequencing task (working memory), token motor task (motor speed), category instances and controlled oral word association test (verbal fluency), symbol coding (attention and processing speed), and Tower of London (executive function). Performances on the six subtests of the BACS were standardized by creating z-scores adjusted for sex at birth (hereafter referred to as sex) and age using norms of Keefe et al. (2004), which were subsequently averaged to create a single composite z-score reflecting global cognitive functioning. Participants missing the BACS assessment (n = 40) or with more than two missing cognitive subscores (n = 1) were excluded from analysis. For participants with ≤2 missing subscores, scores were replaced by the corresponding population mean for that specific domain (n = 9). Finally, three participants (n = 3) were excluded due to outliers on the BACS assessment. This included one participant with intellectual disabilities, one participant with dyslexia and one participant who was not a Dutch native speaker (due to a language barrier).

### Medication use

Use of antipsychotic medication in this study was based on self-reports combined with individual data from the Dutch Foundation for Pharmaceutical Statistics. For a more accurate measure of medication use, self-reports were used as a primary source and were confirmed with the pharmacy records. The Dutch Foundation for Pharmaceutical Statistics collects dispensation data from 99% of community pharmacies in the Netherlands. Using a matching procedure, we received information on included participants concerning the date antipsychotic medication was handed out, including the generic name of the drug, Anatomical Therapeutic Chemical (ATC5) classification, dose per unit (pills or injectables), total number of units prescribed, and daily dose. Data were matched using a pseudonym based on date of birth, name, sex, and postal code. Therefore, data were still available even when a patient collected medication at different pharmacies. As long as the pharmacy shared pseudonyms – which is the case in more than 90% of participating pharmacies, data were available.

Daily dose of antipsychotic medication was converted to olanzapine equivalents (mg/day) (Leucht et al., 2020). Dopamine D_2_ receptor occupancy percentages were estimated based on published PET and SPECT results, following the meta-analytically derived formulas described by Lako et al. (2013). Dopamine D_2_ receptor occupancy could be calculated for amisulpride, aripiprazole, clozapine, haloperidol, olanzapine, quetiapine, risperidone, and paliperidone users (91.3% of participants). Antipsychotics were further categorized into three groups based on their D_2_ receptor affinity: ‘high-affinity antagonists’ if the D_2_ binding affinity Ki < 10 (flupentixol, haloperidol, pimozide, risperidone, zuclopenthixol, sulpiride, paliperidone, penfluridol, amisulpride, and lurasidone); ‘low-affinity antagonists’ for Ki > 10 (olanzapine, clozapine, and quetiapine); and ‘partial D_2_ agonists’ for aripiprazole, brexpiprazole, and cariprazine (Psychoactive Drug Screening Program Ki Database: https://pdsp.unc.edu/databases/kidb.php; Kaar, Natesan, McCutcheon, & Howes, 2020).

### Statistical analysis

Statistical analyses were performed using R version 4.0.3. Block-wise multiple regression analysis, with the BACS composite score as the dependent variable, was used to test several models. Residuals of the models were checked for normality, linearity, and homoscedasticity. The assumption of multicollinearity among all independent variables was not violated (Variance Inflation Factor < 5).

First, the relation between the BACS composite and dopamine D_2_ receptor occupancy was sequentially tested with two models: (1) demographic characteristics (age, sex, and years of education) and symptom severity at testing (PANSS total score) and (2) dopamine D_2_ receptor occupancy added to model 1. Six subsequent exploratory analyses were performed using the same statistical approach for each cognitive domain.

Next, the relation between the BACS composite score and daily dose of antipsychotic in different groups of antipsychotic dopamine D_2_ receptor affinity was tested with three models: (1) demographic characteristics (age, sex, and years of education), symptom severity at testing (PANSS total score), and daily antipsychotic dose (olanzapine equivalents, mg/day); (2) dopamine D_2_ receptor affinity group (low D_2_ receptor affinity; high D_2_ receptor affinity; partial D_2_ receptor agonists) added to model 1; and (3) the interaction between antipsychotic dose * dopamine D_2_ receptor affinity group added to model 2.

Since we analyzed all outcomes twice (dopamine D_2_ receptor occupancy and the interaction between antipsychotic dose and dopamine D_2_ affinity group), we applied a Bonferroni correction to the conventional α-level of 0.05, setting the threshold for statistical significance at 0.025 (0.05/2).

All regression analyses were corrected with IPTW to correct for bias by indication. IPTW was applied as a statistical solution for patient matching to balance potential clinical and sociodemographic differences between the dopamine D_2_ receptor affinity groups. These variables included age, sex, years of education, illness duration, PANSS subscores at testing, and antipsychotic dose at the time of remission in olanzapine equivalents.

## Results

From a total of 287 participants with FEP, data on cognition and medication (type and dose) were sufficiently complete for subsequent analyses. Dopamine D_2_ receptor occupancy could be estimated for 262 participants. Sociodemographic and clinical characteristics are presented in Table 1. Participants had a mean age of 27.9 years (SD = 8.9, median = 25.0, interquartile range (IQR) = 11.0) and consisted of 83 females (29.9%) and 195 males (70.1%). Sociodemographic and clinical characteristics per dopamine D_2_ affinity group are presented in Supplementary Table S1.

**Figure 1.**
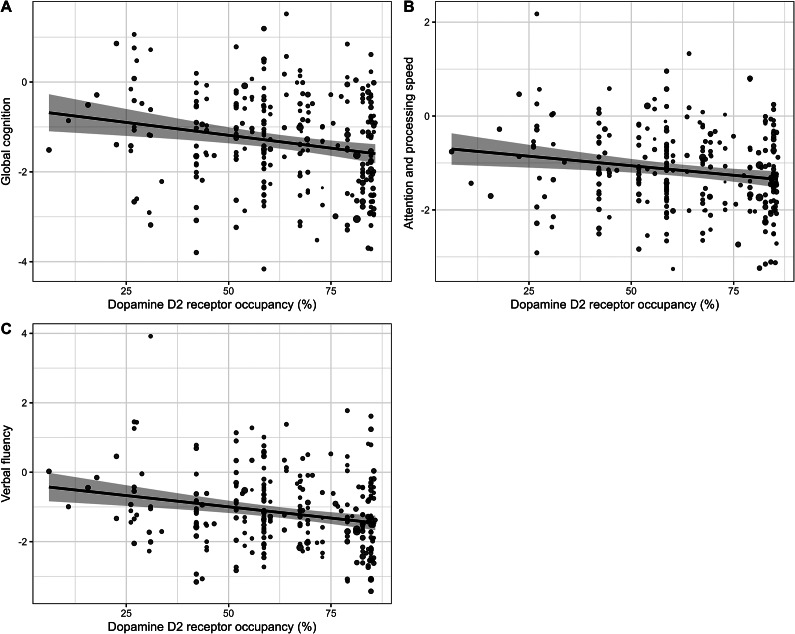
The association between estimated dopamine D_2_ receptor occupancy and (A) global cognitive functioning (BACS composite score); (B) attention and processing speed; and (C) verbal fluency.

The final regression model (n = 262) testing the relation between the BACS composite score and dopamine D_2_ receptor occupancy (corrected for demographics and symptom severity) explained 22.2% of the total variance in global cognitive functioning. Specifically, 18.7% of the variance was explained by the covariates age, sex, years of education, and symptom severity (F(4,257) = 14.80, *p* < 0.001), and an additional 3.5% was explained by the D_2_ receptor occupancy (F(1,256) = 11.58, *p* < 0.001). A higher dopamine D_2_ receptor occupancy was significantly related to lower global cognitive functioning (*β* = −0.18, *p* = 0.0008), as shown in Figure 1A.

Subsequent exploratory analyses for specific subdomains of cognitive functioning, corrected for demographics and symptom severity, demonstrated significant negative associations between dopamine D_2_ receptor occupancy and verbal fluency (*β* = −0.22, *p* = 0.0001) and attention and processing speed (*β* = −0.17, *p* = 0.003), as shown in Figure 1B,C. Detailed statistics on all regression models are provided in Supplementary Tables S2 and S3.

The final regression model testing the relation between the BACS composite score and the interaction between current daily antipsychotic dose and the different dopamine D_2_ receptor affinity groups, explained 26.9% of the total variance in global cognitive functioning. Specifically, 23.2% of the variance was explained by the covariates age, sex, years of education, and symptom severity (F(5,272) = 16.43, *p* < 0.001), and an additional 3.0% was explained by the interaction between antipsychotic dose * D_2_ receptor affinity (F(2,268) = 5.49, *p* = 0.005).

**Figure 2.**
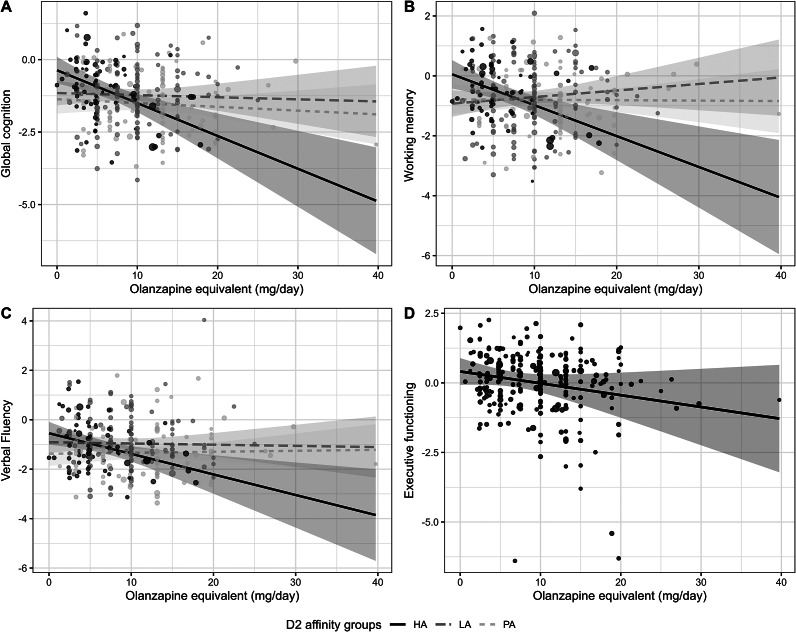
The association between daily antipsychotic dose and (the interaction with) different groups of dopamine D_2_ receptor affinity (high affinity [HA]: continuous; low affinity [LA]: striped; partial agonists [PA]: dotted) with (A) global cognitive functioning (BACS composite score); (B) working memory; (C) verbal fluency; and (D) executive functioning.

The interaction between daily antipsychotic dose and the different groups of antipsychotic dopamine D_2_ receptor affinity was also significant for working memory (R^2^-change = 0.041, F(2,268) = 6.4, *p* = 0.002), but the effect for verbal fluency did not hold after correction for multiple testing (R^2^-change = 0.024, F = (2,268) = 3.59, *p* = 0.029) (Figure 2A–C). All interactions showed the same direction of effects (Figure 2A–C): Users of high-affinity antagonists showed a significantly stronger relationship between high daily antipsychotic dose and low global cognitive functioning, compared to users of low-affinity antagonists and partial agonists (all *β* > 0.40, all *p* < 0.025; Supplementary Tables S4 and S5). While executive functioning did not show a significant interaction effect, the association with current daily antipsychotic dose was statistically significant, irrespective of antipsychotic dopamine D_2_ receptor affinity group (Figure 2D, *β* = −0.17, *p* = 0.0028). Detailed statistics of all regression models are presented in Supplementary Tables S4 and S5.

## Discussion

The current study examined the association between cognitive functioning and dopamine D_2_ receptor occupancy, dopamine D_2_ receptor affinity, and antipsychotic daily dose in 278 participants recently remitted from an FEP. Our results demonstrated that dopamine D_2_ receptor occupancy was negatively related to global cognitive functioning, verbal fluency, and attention and processing speed, indicating that a higher D_2_ receptor occupancy was related to lower cognitive functioning. In addition, the interaction between the current daily dose of antipsychotic and the different groups of antipsychotic dopamine D_2_ receptor affinity was not only significant for global cognitive functioning, but also for working memory, and at the trend level for verbal fluency. Users of high-affinity antagonists (e.g. haloperidol, risperidone, amisulpride) showed a significantly stronger relationship between high daily antipsychotic dose and low global cognitive functioning, compared to users of low-affinity antagonists and partial agonists. While executive functioning did not show a significant interaction effect, lower executive functioning was related to higher daily antipsychotic doses, irrespective of antipsychotic dopamine D_2_ receptor affinity.

The variance in global cognitive functioning in FEP is considerable (Catalan et al., 2024), and generally poorly explained. Multiple studies have found that – even when combining demographic and clinical characteristics, including proxies of premorbid functioning – no more than 30% of the variance is typically accounted for (Amoretti et al., 2021; Cuesta et al., 2015; González-Blanch et al., 2008; Lutgens, Lepage, Iyer, & Malla, 2014). Other important predictors such as age, sex (Carruthers, Van Rheenen, Karantonis, & Rossell, 2022), negative symptom severity (Au-Yeung et al., 2023), illness insight (Subotnik et al., 2020), and polygenic risk scores (Hubbard et al., 2016), individually explain less than 10% of the variance in neurocognitive functioning. For instance, Lutgens et al. (2014) found that socio-economic status uniquely explained only 4% of the variance in global cognitive functioning in a comparable sample of 269 participants with FEP (average global cognition z = −1.48) (Lutgens et al., 2014). Given the substantial unexplained variance in cognitive functioning in FEP, and recognizing that cognitive impairment is significantly associated with social and occupational functioning, as well as quality of life (Cowman et al., 2021; Fett et al., 2011; Tolman & Kurtz, 2012), the 3.5% of variance explained by D_2_ receptor occupancy in our study contributes a new piece of the puzzle and can be considered of clinical importance when selecting an antipsychotic drug. It is plausible that in samples with greater cognitive impairment, even stronger effects might emerge. Importantly, many factors, such as demographic and genetic background, cannot be targeted and modified to improve cognition, but the type and dose of medication are factors that can be considered when treating individuals with FEP.

While the association of cognitive functioning with antipsychotic dose and receptor affinity has been reported before (Baitz et al., 2012; Singh et al., 2022), their interactive effect in combination with D_2_ receptor occupancy in a large sample of people remitted from an FEP is new and suggests that antipsychotic medication may affect cognitive functioning via D_2_ receptor occupancy. In parallel to our current finding, our group recently observed more speech deviations (De Boer et al., 2020) and more severe negative symptoms (de Beer et al., 2024) in users of high dopamine D_2_ affinity antipsychotics compared to users of partial agonists or low-affinity antagonists, and previous research demonstrated a decline in cognitive functioning in individuals prescribed risperidone/paliperidone, both high dopamine D2 affinity antagonists (Allott et al., 2023). Furthermore, several studies concluded that high daily doses of antipsychotics had deleterious effects on verbal fluency and processing speed (Élie et al., 2010; Rehse et al., 2016; Woodward et al., 2007). However, the ratio of receptors that are occupied by antipsychotics is dependent on both receptor affinity and antipsychotic dose. Studies that have related dopamine D_2_ receptor occupancy (as derived from PET or plasma levels of antipsychotics) to cognition, also reported that high levels of occupancy were related to poor global cognitive functioning and attention in older individuals with long-term SSD (Sakurai et al., 2013; Uchida et al., 2009). More recent studies suggested lower cognitive functioning and hyperprolactinemia even above a threshold of 67% dopamine D_2_ receptor occupancy in people with long-term SSD (Iwata et al., 2016; Kusudo et al., 2022). The current findings suggest that high antipsychotic dopamine D_2_ receptor occupancy may have considerable negative effects on neurocognitive functioning also in young individuals with FEP. This underscores the importance of careful determination of the antipsychotic medication type in combination with the lowest effective dose, early on in psychosis treatment.

The dosage of antipsychotic medication in the current sample (median 9.48 ± IQR 8.16 mg olanzapine) follows the recommendations of several guidelines to prescribe the lowest effective dose, especially in people with FEP (Buchanan et al., 2010; Correll et al., 2022). Findings of a recent study on medication strategies in the Netherlands indicated that 34% of the clinicians already began dose reduction approximately 4 months after remission from an FEP (Kikkert et al., 2022). In our current data, negative effects of high D_2_ occupancy were evident even at relatively low doses, suggesting that, even in a relatively low-dose range, negative effects of higher doses can be observed, and not only dose reduction, but also the type of antipsychotic impacts cognition. Recent meta-analyses also concluded that haloperidol is among the worst-performing antipsychotics with regard to cognitive functioning, shown for both global composite scores and all studied cognitive domains (Baldez et al., 2021; Feber et al., 2024). The current study extends these findings to antipsychotics with strong dopamine affinity as a group and provides important leads for choosing antipsychotic drugs in individuals with FEP, as dopamine D_2_ receptor affinity may affect cognition in young people, even at low doses.

Current findings are of clinical importance, with implications for prescribing antipsychotics to individuals with an FEP. All antipsychotics have their own specific side-effect profiles, and the process of shared decision-making is the preferred strategy to choose the drug with the patient’s most favorable side effect profile (Van Dijk et al., 2018). During this process, patients need to be informed about the potential negative effects of high dopamine affinity drugs on cognition. When patients prioritize the impact on cognitive functioning, medication with low D_2_ receptor affinity or partial agonists may be preferred. While the European Psychiatric Association guidelines recommend second-generation antipsychotics based on their favorable cognitive profile (Vita et al., 2022), this recommendation should be tailored to D_2_ receptor affinity, as several atypical drugs also have high dopamine affinity (i.e. risperidone, paliperidone, sulpiride, amisulpride).

### Strengths and limitations

The main result of this study (i.e. the association between high D_2_ receptor occupancy and affinity and low global cognitive functioning) is based on a well-powered analysis (n = 278). Furthermore, participants were included shortly after diagnosis and had all achieved symptomatic remission before testing, so symptom severity was relatively low. Therefore, the potential confounding effect of other factors that may impact cognitive functioning, such as illness duration and severity of psychotic symptoms, was limited. However, participants were not randomized to the type of medication, but all regression analyses were corrected using IPTW as a statistical method that adjusts for differences in clinical and sociodemographic characteristics to limit the effects of bias by indication. While IPTW balances observed characteristics across the three D_2_ affinity groups, the findings may potentially still be influenced by unmeasured characteristics. Participants were recruited from 26 mental healthcare institutions throughout the Netherlands, covering different patient groups and practices, which may increase the generalizability of our findings. Another strength is that we used pharmacy dispensation data to confirm antipsychotic medication use. Most studies examining the association between the type and dose of antipsychotic medication and cognitive functioning are hampered by poor measures of medication use (i.e. self-report only), while low medication adherence has been reported in SSD (Roberts & Velligan, 2011) and patients’ self-report of medication use often overestimates adherence (Jónsdóttir et al., 2010). While still not flawless, pharmacy dispensation data are closer to the actual dose of medication taken than self-reports only. However, our study does not permit conclusions regarding causal relationships between medication use and cognitive functioning, due to its cross-sectional design. There have been some antipsychotic dose-reduction studies with initial positive results for cognitive improvement (Singh et al., 2022; Takeuchi et al., 2013; Zhou et al., 2018), but those studies were too small to stratify their analyses based on antipsychotic type. For that purpose, we need larger samples and confirmation in longitudinal studies in which patients are switched from high- to low-affinity drugs or to partial agonists. Furthermore, despite recent evidence indicating an association between anticholinergic burden and cognitive function in psychosis (Mancini et al., 2025), the effect of anticholinergic burden on cognitive functioning was not assessed in the current study. However, as cholinergic innervation of the brain declines steeply with age, anticholinergic burden is expected to be more troublesome for the cognitive functioning of older people than for our participants with an average age of 28 years (Orlando et al., 2023).

## Conclusion

The current study found negative effects of high dopamine D_2_ receptor occupancy and high D_2_ receptor affinity antipsychotics on cognitive functioning in individuals remitted from an FEP, even at a low dose. This underscores the importance of the careful selection of the optimal antipsychotic type and dose already at the start of treatment in FEP, as medication with low dopamine D_2_ receptor affinity or partial agonists may minimize the negative impact on cognition.

## Supporting information

10.1017/S0033291725102900.sm001 Oomen et al. supplementary material Oomen et al. supplementary material

## Supplementary material

The supplementary material for this article can be found at http://doi.org/10.1017/S0033291725102900.
