# Supplementary material for: Negative association between cognitive functioning and antipsychotic D2 receptor occupancy, affinity, and dose after first episode psychosis
Source: Psychol Med. 2026 Jan 2;56:e5. doi: 10.1017/S0033291725102900 (PMC13092990; doi:10.1017/S0033291725102900)
Supplement: Oomen et al. supplementary material [file S0033291725102900sup001.docx]

**Supplementary materials**

**Table S1. Sociodemographic and clinical characteristics per dopamine D2 affinity group (HA = high D2 receptor affinity; LA = low D2 receptor affinity; PA = partial D2 receptor agonists).**

|  | **HA (N=70)** | **LA (N=124)** | **PA (N=84)** | **Overall (N=278)** |
| --- | --- | --- | --- | --- |
| **Age (years)** |  |  |  |  |
| Mean (SD) | 27.9 (10.2) | 27.8 (8.12) | 28.2 (8.87) | 27.9 (8.86) |
| Median [IQR] | 23.5 [10.0] | 25.0 [11.0] | 25.0 [11.3] | 25.0 [11.0] |
| **Sex at birth** |  |  |  |  |
| Male | 52 (74.3%) | 87 (70.2%) | 56 (66.7%) | 195 (70.1%) |
| Femal | 18 (25.7%) | 37 (29.8%) | 28 (33.3%) | 83 (29.9%) |
| **Years of education** |  |  |  |  |
| Mean (SD) | 13.9 (2.81) | 13.8 (2.34) | 14.0 (2.27) | 13.9 (2.44) |
| Median [IQR] | 14.0 [3.00] | 14.0 [1.03] | 14.0 [1.00] | 14.0 [1.09] |
| **Ilness duration (months)** |  |  |  |  |
| Mean (SD) | 13.7 (24.6) | 8.46 (5.29) | 13.7 (24.9) | 11.4 (18.8) |
| Median [IQR] | 7.00 [4.00] | 7.00 [5.00] | 9.00 [7.25] | 8.00 [5.00] |
| **PANSS positive symptoms** |  |  |  |  |
| Mean (SD) | 8.74 (2.17) | 9.13 (2.65) | 9.01 (2.53) | 9.00 (2.50) |
| Median [IQR] | 8.00 [3.00] | 8.00 [4.00] | 8.00 [3.00] | 8.00 [3.75] |
| **PANSS negative symptoms** |  |  |  |  |
| Mean (SD) | 12.5 (4.45) | 12.5 (4.08) | 11.4 (4.16) | 12.2 (4.21) |
| Median [IQR] | 12.0 [6.75] | 12.0 [6.00] | 10.0 [6.00] | 11.0 [6.00] |
| **PANSS general symptoms** |  |  |  |  |
| Mean (SD) | 22.8 (4.90) | 23.4 (5.56) | 23.3 (5.53) | 23.2 (5.38) |
| Median [IQR] | 22.0 [6.75] | 22.0 [7.00] | 23.0 [7.00] | 22.0 [7.00] |
| **PANSS totalscore** |  |  |  |  |
| Mean (SD) | 44.0 (8.91) | 45.0 (9.90) | 43.7 (10.2) | 44.4 (9.72) |
| Median [IQR] | 42.5 [12.5] | 43.0 [14.3] | 41.5 [11.3] | 42.0 [14.0] |
| **Antipsychotic daily dose (mg olanzapine)** |  |  |  |  |
| Mean (SD) | 5.83 (3.87) | 9.73 (4.76) | 12.0 (6.63) | 9.45 (5.68) |
| Median [IQR] | 4.76 [3.87] | 10.0 [6.28] | 13.2 [6.78] | 9.48 [8.16] |
| **Dopamine D2 receptor occupancy (%)** |  |  |  |  |
| Mean (SD) | 64.1 (10.7) | 49.9 (15.3) | 83.4 (2.55) | 63.4 (18.7) |
| Median [IQR] | 67.2 [13.7] | 51.8 [16.5] | 84.8 [2.02] | 65.4 [30.9] |
| Missing | 14 (20.0%) | 0 (0%) | 2 (2.4%) | 16 (5.8%) |
| **Time since remission until cognitive testing (days)** |  |  |  |  |
| Mean (SD) | 133 (53.3) | 133 (44.1) | 148 (59.9) | 138 (51.9) |
| Median [IQR] | 126 [59.0] | 130 [62.5] | 142 [70.0] | 132 [64.3] |
| Missing | 1 (1.4%) | 1 (0.8%) | 0 (0%) | 2 (0.7%) |
| **BACS composite, Z-score** |  |  |  |  |
| Mean (SD) | -1.10 (1.14) | -1.29 (1.18) | -1.55 (1.17) | -1.32 (1.18) |
| Median [IQR] | -1.02 [1.33] | -1.20 [1.74] | -1.67 [1.81] | -1.26 [1.71] |
| **Verbal memory, Z-score** |  |  |  |  |
| Mean (SD) | -0.36 (1.06) | -0.81 (1.15) | -0.85 (1.11) | -0.71 (1.13) |
| Median [IQR] | -0.37 [1.35] | -0.72 [1.58] | -0.83 [1.34] | -0.71 [1.41] |
| **Working memory, Z-score** |  |  |  |  |
| Mean (SD) | -0.60 (1.05) | -0.79 (1.25) | -0.89 (0.993) | -0.78 (1.13) |
| Median [IQR] | -0.57 [1.70] | -0.73 [1.96] | -0.99 [1.29] | -0.73 [1.79] |
| **Motor speed, Z-score** |  |  |  |  |
| Mean (SD) | -0.75 (1.30) | -0.86 (1.22) | -0.87 (1.45) | -0.83 (1.31) |
| Median [IQR] | -0.67 [1.80] | -0.77 [1.59] | -0.67 [1.82] | -0.67 [1.64] |
| **Verbal fluency, Z-score** |  |  |  |  |
| Mean (SD) | -1.06 (1.04) | -0.88 (1.14) | -1.21 (1.09) | -1.02 (1.10) |
| Median [IQR] | -1.25 [1.48] | -1.07 [1.45] | -1.34 [1.28] | -1.16 [1.46] |
| **Attention & processing speed, Z-score** |  |  |  |  |
| Mean (SD) | -1.26 (0.992) | -1.15 (0.851) | -1.39 (0.876) | -1.25 (0.898) |
| Median [IQR] | -1.13 [1.23] | -1.19 [0.991] | -1.44 [0.900] | -1.26 [1.08] |
| **Executive function, Z-score** |  |  |  |  |
| Mean (SD) | 0.06 (1.16) | -0.17 (1.13) | -0.33 (1.27) | -0.16 (1.19) |
| Median [IQR] | 0.09 [1.13] | 0.077 [1.10] | 0.08 [1.27] | 0.08 [1.10] |

LA, low D2 receptor affinity; HA, high D2 receptor affinity; PA, partial D2 receptor agonists; PANSS, Positive and Negative Syndrome Scale; BACS, Brief Assessment of Cognition in Schizophrenia.

**Table S2. Model summary of HMR analyses of cognition and dopamine D_2_ receptor occupancy after controlling for demographics and symptom severity.**

| N=262 | **Model 1**  Demographics (age, gender, YoE) and symptom severity (PANSS) | | | **Model 2**  Dopamine D_2_ receptor occupancy | | |  |
| --- | --- | --- | --- | --- | --- | --- | --- |
| **Cognitive domain** | R^2^ change | F-statistics | p-value | R^2^ change | F-statistics | p-value | R^2^ total |
| Global cognition | 0.187 | F(4,257)=14.80 | **<0.0001** | 0.035 | F(1,256)=11.58 | **0.0008** | 0.222 |
| Verbal memory | 0.176 | F(4,257)=13.74 | **<0.0001** | 0.008 | F(1,256)=2.67 | 0.10 | 0.185 |
| Motor speed | 0.090 | F(4,257)=6.34 | **0.0001** | 0.011 | F(1,256)=3.17 | 0.076 | 0.101 |
| Working memory | 0.080 | F(4,257)=5.55 | **0.0003** | 0.002 | F(1,256)=0.43 | 0.51 | 0.081 |
| Verbal fluency | 0.064 | F(4,257)=4.38 | **0.0019** | 0.052 | F(1,256)=15.08 | **0.0001** | 0.116 |
| Attention & processing speed | 0.080 | F(4,257)=5.56 | **0.0003** | 0.031 | F(1,256)=8.83 | **0.0032** | 0.110 |
| Executive functioning | 0.090 | F(4,257)=6.35 | **0.0001** | 0.009 | F(1,256)=2.42 | 0.12 | 0.098 |

PANSS, Positive and Negative Syndrome Scale; YoE, Years of Education

**Table S3. Relation (β, p-value) between dopamine D_2_ receptor occupancy and global cognition and cognitive subdomains in FEP**

| **Cognitive domain** | Global cognition | | Verbal memory | | Motor speed | | Working memory | | Verbal fluency | | Attention & processing speed | | Executive functioning | |
| --- | --- | --- | --- | --- | --- | --- | --- | --- | --- | --- | --- | --- | --- | --- |
| N=262 | β | p-value | β | p-value | β | p-value | β | p-value | β | p-value | β | p-value | β | p-value |
| Dopamine D2R occupancy | -0.18 | **0.0008** | -0.09 | 0.10 | -0.10 | 0.076 | -0.04 | 0.51 | -0.22 | **0.0001** | -0.17 | **0.0032** | -0.09 | 0.12 |
| Gender | -0.06 | 0.63 | -0.39 | **0.0021** | 0.11 | 0.41 | -0.08 | 0.54 | 0.23 | 0.075 | -0.20 | 0.13 | -0.36 | **0.0052** |
| Years of education | 0.26 | **<0.0001** | 0.26 | **<0.0001** | 0.16 | **0.0086** | 0.17 | **0.0069** | 0.14 | **0.0230** | 0.15 | **0.0167** | 0.12 | 0.056 |
| PANSS total | -0.31 | **<0.0001** | -0.27 | **<0.0001** | -0.21 | **0.0004** | -0.20 | **0.0011** | -0.09 | 0.11 | -0.20 | **0.0006** | -0.17 | **0.0033** |

FEP, First Episode Psychosis; D2R, D_2_ receptor; PANSS, Positive and Negative Syndrome Scale.

The table displays standardized (β) regression coefficients and p-values.

**Table S4. Model summary of HMR analyses of cognition and daily dose of antipsychotic medication in different groups of dopamine D_2_ receptor affinity, after controlling for demographics and symptom severity**

| N=278 | **Model 1**  Demographics (age, gender, YoE), symptom severity (PANSS) and daily dose of antipsychotic medication | | | **Model 2**  Dopamine D_2_ receptor affinity group (low D_2_ affinity; high D_2_ affinity; partial D_2_ agonists) | | | **Model 3**  Daily dose of antipsychotic medication * dopamine D_2_ receptor affinity group | | |  |
| --- | --- | --- | --- | --- | --- | --- | --- | --- | --- | --- |
| **Cognitive domain** | R^2^ change | F-statistics | p-value | R^2^ change | F-statistics | p-value | R^2^ change | F-statistics | p-value | R^2^ total |
| Global cognition | 0.232 | F(5,272)=16.43 | **<0.0001** | 0.007 | F(2,270)=1.20 | 0.30 | 0.030 | F(2,268)=5.49 | **0.0046** | 0.27 |
| Verbal memory | 0.184 | F(5,272)=12.29 | **<0.0001** | 0.020 | F(2,270)=3.47 | 0.03 | 0.007 | F(2,268)=1.14 | 0.32 | 0.21 |
| Motor speed | 0.113 | F(5,272)=6.96 | **<0.0001** | 0.001 | F(2,270)=0.21 | 0.81 | 0.009 | F(2,268)=1.40 | 0.25 | 0.12 |
| Working memory | 0.106 | F(5,272)=6.44 | **<0.0001** | 0.001 | F(2,270)=0.10 | 0.90 | 0.041 | F(2,268)=6.49 | **0.0019** | 0.15 |
| Verbal fluency | 0.082 | F(5,272)=4.88 | **0.0003** | 0.016 | F(2,270)=2.44 | 0.089 | 0.024 | F(2,268)=3.59 | **0.0288** | 0.12 |
| Attention & processing speed | 0.082 | F(5,272)=4.87 | **0.0003** | 0.012 | F(2,270)=1.81 | 0.17 | 0.014 | F(2,268)=2.10 | 0.13 | 0.11 |
| Executive functioning | 0.128 | F(5,272)=7.95 | **<0.0001** | 0.002 | F(2,270)=0.27 | 0.76 | 0.001 | F(2,268)=0.22 | 0.80 | 0.13 |

PANSS, Positive and Negative Syndrome Scale; YoE, Years of Education

**Table S5. Relation (β, p-value) between daily dose of antipsychotic medication and different groups of dopamine D_2_ receptor affinity and global cognition and cognitive subdomains in FEP**

| **Cognitive domain** | Global cognition | | Verbal memory | | Motor speed | | Working memory | | Verbal fluency | | Attention & processing speed | | Executive functioning | |
| --- | --- | --- | --- | --- | --- | --- | --- | --- | --- | --- | --- | --- | --- | --- |
| N=278 | β | p-value | β | p-value | β | p-value | β | p-value | β | p-value | β | p-value | β | p-value |
| Daily dose of antipsychotic | -0.17 | **0.0028** | -0.11 | 0.056 | -0.11 | 0.059 | -0.07 | 0.26 | -0.10 | 0.010 | -0.10 | 0.11 | -0.17 | **0.0028** |
| *Dopamine D2R affinity group* |  |  |  |  |  |  |  |  |  |  |  |  |  |  |
| Low affinity | -0.003 | 0.98 | -0.32 | **0.0220** | 0.09 | 0.55 | -0.03 | 0.82 | 0.18 | 0.22 | 0.21 | 0.14 | -0.03 | 0.86 |
| Partial agonist | -0.19 | 0.21 | -0.38 | **0.0160** | 0.10 | 0.56 | -0.08 | 0.65 | -0.12 | 0.48 | -0.01 | 0.95 | -0.11 | 0.50 |
| *Daily dose * D2R affinity group* |  |  |  |  |  |  |  |  |  |  |  |  |  |  |
| Low affinity | 0.51 | **0.0024** | 0.15 | 0.39 | 0.30 | 0.10 | 0.63 | **0.0006** | 0.40 | **0.025** | 0.37 | **0.0429** | 0.01 | 0.94 |
| Partial agonist | 0.48 | **0.0030** | 0.25 | 0.14 | 0.19 | 0.27 | 0.51 | **0.0036** | 0.45 | **0.010** | 0.27 | 0.12 | 0.09 | 0.59 |
| Gender | -0.10 | 0.40 | -0.42 | **0.0007** | 0.10 | 0.46 | -0.10 | 0.46 | 0.16 | 0.23 | -0.19 | 0.14 | -0.38 | **0.0025** |
| Years of education | 0.27 | **<0.0001** | 0.24 | **<0.0001** | 0.18 | **0.0036** | 0.19 | **0.0025** | 0.16 | **0.007** | 0.14 | **0.022** | 0.14 | **0.021** |
| PANSS total | -0.29 | **<0.0001** | -0.25 | **<0.0001** | -0.20 | **0.0008** | -0.20 | **0.0007** | -0.11 | 0.056 | -0.18 | **0.0026** | -0.15 | **0.0099** |

FEP, First Episode Psychosis; D2R, D_2_ receptor; PANSS, Positive and Negative Syndrome Scale.

The table displays standardized (β) regression coefficients and p-values.
